# FDTD Analysis of Hotspot-Enabling Hybrid Nanohole-Nanoparticle Structures for SERS Detection

**DOI:** 10.3390/bios12020128

**Published:** 2022-02-17

**Authors:** Juan Gomez-Cruz, Yazan Bdour, Kevin Stamplecoskie, Carlos Escobedo

**Affiliations:** 1 Department of Chemical Engineering, Queen’s University, 19 Division St., Kingston, ON K7L 3N6, Canada; j.gomezcruz@queensu.ca (J.G.-C.); 16yb6@queensu.ca (Y.B.); 2 Department of Chemistry, Queen’s University, 90 Bader Lane, Kingston, ON K7L 3N6, Canada; kevin.stamplecoskie@queensu.ca

**Keywords:** metallic nanoparticles, nanohole arrays, FDTD, SERS-active structures, hotspots

## Abstract

Metallic nanoparticles (MNPs) and metallic nanostructures are both commonly used, independently, as SERS substrates due to their enhanced plasmonic activity. In this work, we introduce and investigate a hybrid nanostructure with strong SERS activity that benefits from the collective plasmonic response of the combination of MNPs and flow-through nanohole arrays (NHAs). The electric field distribution and electromagnetic enhancement factor of hybrid structures composed of silver NPs on both silver and gold NHAs are investigated via finite-difference time-domain (FDTD) analyses. This computational approach is used to find optimal spatial configurations of the nanoparticle positions relative to the nanoapertures and investigate the difference between Ag-NP-on-Ag-NHAs and Ag-NP-on-Au-NHAs hybrid structures. A maximum G_SERS_ value of 6.8 × 10^9^ is achieved with the all-silver structure when the NP is located 0.5 nm away from the rim of the NHA, while the maximum of 4.7 × 10^10^ is obtained when the nanoparticle is in full contact with the NHA for the gold-silver hybrid structure. These results demonstrate that the hybrid nanostructures enable hotspot formation with strong SERS activity and plasmonic enhancement compatible with SERS-based sensing applications.

## 1. Introduction

Metallic nanostructures that support surface plasmon resonance (SPR) have been extensively researched in the past decade and employed in several sensing and biosensing applications, including cell analysis [1], the detection and quantification of infectious diseases [2,3,4], and cancer biomarker quantification [5]. SPR is based on the collective oscillation of conduction electrons, which promotes an enhancement of the electromagnetic field at the metal-dielectric interface [6]. The plasmonic signal arising from the resonance is highly sensitive to changes in refractive index (RI) at the interface. Minute changes in RI cause a resonance shift in its reflection or transmission spectra, enabling not only the detection but also the quantification of analytes. In the context of (bio)sensing, this phenomenon has found applications in techniques such as SPR spectroscopy [1,2,7,8], SPR imaging [3,4,9], and surface-enhanced Raman scattering (SERS) spectroscopy [10,11,12]. SERS is a light scattering-based technique that enables the detection of chemical and biological analytes in a label-free fashion owing to the inherent and unique vibrational modes of the molecules. SERS-active nanostructures enhance the vibrational excitation of molecules due to a confined enhancement of electromagnetic field at the metal surface, so-called “hotspots”, which translates into a stronger signal due to the high level of light scattering from the sample [13,14]. The high specificity and low absorption of electromagnetic radiation in the VIS/NIR spectrum by water [15] make this technique suitable for analyte detection in fluids [10]. SERS has been demonstrated to boost the Raman sensitivity by several orders of magnitude, even in small amounts of analytes.

Metallic nanoparticles (MNPs), in particular, are commonly used to create SERS-active substrates due to their confined localized SPR (LSPR) upon wavelength-specific light excitation [16]. Despite the multiple advantages offered by MNP-based SERS substrates, including their low cost and ease of fabrication, reproducibility still remains a challenge [17,18]. The nanoplasmonic capabilities of nanohole arrays (NHAs) have been theoretically predicted and demonstrated through FDTD simulations [19,20] and experimentation [3,21]. In more recent work, NHAs have been demonstrated as plausible and highly reproducible [22] SERS substrates. Additionally, the plasmonic properties of NHA structures can be easily manipulated by tailoring their geometric parameters, providing a uniform enhancement over a large surface area [23]. However, NHAs still cannot achieve SERS enhancement factors (EF) comparable to the values obtained on MNP-based SERS substrates [24,25,26,27]. Recent research has focused on combining the outstanding EF of MNP-based assemblies with the reproducibility of metallic NHAs. Experimental attempts to combine these nanostructures have resulted in promising SERS-active substrates for producing higher SERS EFs than 2D NP assemblies, nanocups, or NHAs individually [28,29]. However, a better understanding of the hotspot generation and plasmonic interaction of the nanostructures is needed to unleash their maximum SERS potential.

In this work, we investigate the electromagnetic field enhancement achieved by the combination of two nanoplasmonic-enabling structures, metallic nanoparticles and nanohole arrays, as a hybrid structure for a potential SERS substrate with hot spots. The plasmonic characteristics of the proposed hybrid SERS-active nanostructure are investigated through the finite-difference time-domain (FDTD) method. A panel of simulations for Ag nanoparticles in both Ag and Au NHA structures was utilized to find the optimal spatial configurations of the NPs in the vicinity of the NHAs, considering the creation of hotspots that ultimately result in a Raman enhancement compatible with SERS applications. Even when the proposed hybrid nanostructure may not enhance the reproducibility of SERS substrates per se, the proximity and relative position of the metallic NP to the surfaces and rims of the nanoholes result in a great enhancement in the electric field (e-field). The proposed configurations may be achieved, in praxis, through electrostatic or electrohydrodynamic means, in combination with surface functionalization approaches [29,30,31]. The study is focused on the electromagnetic enhancement factor, since it represents the main contribution to the intensification of the Raman signal, as opposed to the chemical enhancement [32].

## 2. Materials and Methods

**Figure 1:**
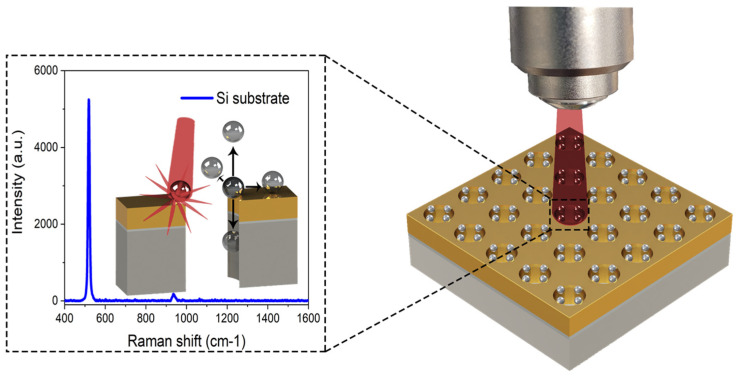
Schematic representation of the hybrid SERS-active nanostructures.

Figure 1 shows a 3D computational model of the hybrid SERS-active nanostructures. The metallic NHAs are decorated with metallic nanoparticles for the generation of hotspots for the enhancement of the Raman signal. The simulated nanostructures combine spherical silver nanoparticles (Ag-NPs) with 50 nm diameters with flow-through, periodic NHA structures of 200 nm diameter and 400 nm periodicity. The NHA structures comprise a 200 nm layer of silicon nitride (Si_3_N_4_), a 5 nm adhesion layer of chrome (Cr), and a 100 nm layer of gold (Au-NHA) or silver (Ag-NHAs).

### 2.1. FDTD Simulations

**Figure 2:**
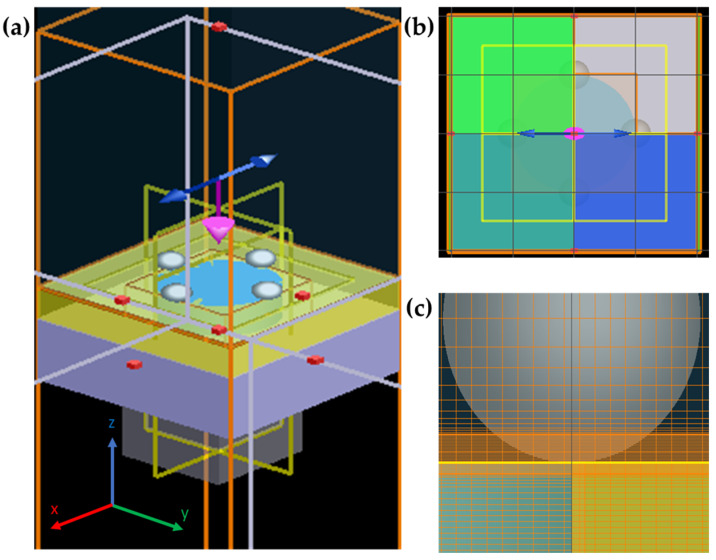
FDTD simulation of the hybrid SERS active nanostructure. (**a**) Perspective view of the simulation environment showing the light polarization in the *x*-direction. (**b**) Symmetric and antisymmetric boundary conditions were set for the *x*- and *y* directions, respectively, and a perfectly matched layer (PML) was set in the *z*-direction. (**c**) Override 3 nm mesh in the *x*- and *y*-axes and 0.2 nm mesh in the *z*-axis were set at the NHA and NP interface to improve the accuracy of the simulations in that region.

Different computational methods are routinely used to solve Maxwell’s equations to analyze and interpret the interaction of light with metallic nanostructures. The finite-difference time-domain (FDTD) method, in particular, is a useful tool to study that interaction and the consequent enhancement of the electromagnetic field at the interface with a dielectric, in proximity to other metallic nanostructures, or in the vicinity of the nanostructure per se, as it allows the non-uniform meshing of the domain to simulate the near- and far-field distribution [33]. For this study, a three-dimensional analysis using Lumerical software (Lumerical FDTD Solutions) was used to simulate the distribution of the near-field electromagnetic field of different configurations involving metallic NPs, films, and nanoholes, as discussed in Section 2.2. The e-field distribution originating from the plasmonic interaction of the light with the nanostructures was recorded at 735 nm using an *x*-polarized plane-wave Total-Field Scattered-Field excitation source centered at a wavelength of 785 nm and a width of 25 nm, as shown in Figure 2a. A plane-wave excitation source was selected under the assumptions that (1) the area of analysis would be smaller than the diameter of the laser beam and that (2) a laser beam could be considered a plane wave within the Rayleigh range [34]. Symmetric and antisymmetric boundary conditions were set for the *x*- and *y*-directions, respectively, and a perfectly matched layer (PML) was set in the *z*-direction, as shown in Figure 2b. The physical properties of water were used for the medium inside the hole and the surroundings. The dielectric permittivity of the metals and the refractive indices of the Si_3_N_4_ and water used in the simulations were obtained from the literature [35,36,37]. An override mesh was used at the interface of the nanoholes and the nanoparticles, for those configurations that involved contact, to improve the simulation accuracy and facilitate the visualization of the electric field distribution at hotspot locations. The sample configuration shown in Figure 2c shows an override mesh with a width of 3 nm in the *x* and *y* axes and 0.2 nm in the *z*-axis at the aforementioned interface. An iterative test was used to identify the element size, which, through numerical result convergence, produced accurate simulations for a specific configuration in the least amount of simulation time possible. The best override mesh size was found to be 3 nm in the *x*- and *y*-axes and 0.2 nm in the *z*-axis.

A time-averaged e-field intensity distribution of the electromagnetic enhancement was calculated by considering the simplified equation for the electromagnetic enhancement factor for a SERS substrate [38,39,40], $G_{\mathrm{SERS}}\approx \;{\left|E_{\mathrm{loc}}\left(\omega_{\mathrm{exc}}\right)/E_{\mathrm{inc}}\left(\omega_{\mathrm{exc}}\right)\right|}^{4}$, where $E_{\mathrm{loc}}\left(\omega_{\mathrm{exc}}\right)$ and $E_{\mathrm{inc}}\left(\omega_{\mathrm{exc}}\right)$ are the amplitudes of the local and the incident electric field, respectively, at the excitation light source angular frequency, $\omega_{\mathrm{exc}}$.

### 2.2. Simulated NP-NHA Hybrid Nanostructure Configurations

**Figure 3:**
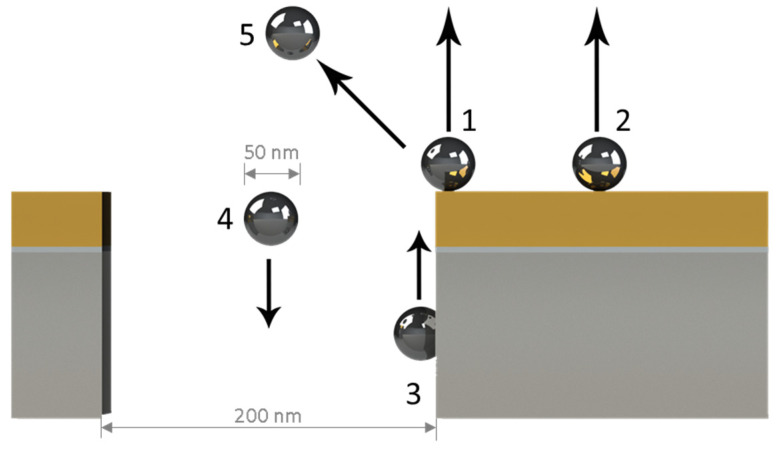
Simulation configurations for different positions of NPs on the NHA surface. In Configuration 1, the Ag-NPs are located on the surface at the rim of the nanohole. In Configuration 2, the NPs are located 150 nm away from the center of the nanohole on the surface of the metal. In Configuration 3, the NPs are in contact with the nanohole’s inner wall. In Configuration 4, the particles are at the center of the nanohole. In Configuration 5, the particles are placed on the edge of the nanohole at 45 degrees. For each configuration, the distance between the NPs and the surface of the nanoholes is varied along the *z*-axis, except in Configuration 5, where the particles are moved away from the edge at 45 degrees.

The electromagnetic field enhancement was evaluated for five different configurations that considered different Ag-NP positions relative to the NHAs and a subsequent variation in the *z*-direction, as shown in Figure 3. Each configuration involved both Au-NHAs and Ag-NHAs. Four NPs were considered to ensure asymmetry relative to a cross-sectional *x–z* plane. Configuration 1 corresponded to Ag-NPs positioned on the surface at the rim of the nanohole, with a concentric alignment between the perimeter and the center of the NP in an *x–z* or *y–z* plane view. In Configuration 2, the NPs were located 150 nm away from the center of the nanohole on the surface of the metal. In Configuration 3, the NPs were in contact with the inner wall of the nanoholes. In Configuration 4, the particles were placed at the center of the nanohole. Finally, in Configuration 5, the particles were positioned symmetrically at the edge of the nanohole. For this last configuration, the position of the particles was not varied along the *z*-axis but along a 45-degree linear direction. For configurations 1 and 2, the distances between the NPs and the surface of the nanoholes used for the simulations were 0 nm, 1 nm, 2 nm, 3 nm, 4 nm, 5 nm, and 10 nm. For configurations 3 and 4, the NPs were displaced 20 nm above and below the nanohole rim along the *z*-axis.

The electromagnetic enhancement of Ag-NHAs and Au-NHAs was evaluated without NPs in order to understand the individual contribution of the NHAs as a SERS structure. Additionally, both Ag and Au flat metallic slabs were evaluated with and without Ag-NPs to investigate the contribution of the NPs without the presence of nanoholes.

## 3. Results and Discussion

### 3.1. Metallic Flats, NHAs, and NPs

**Figure 4:**
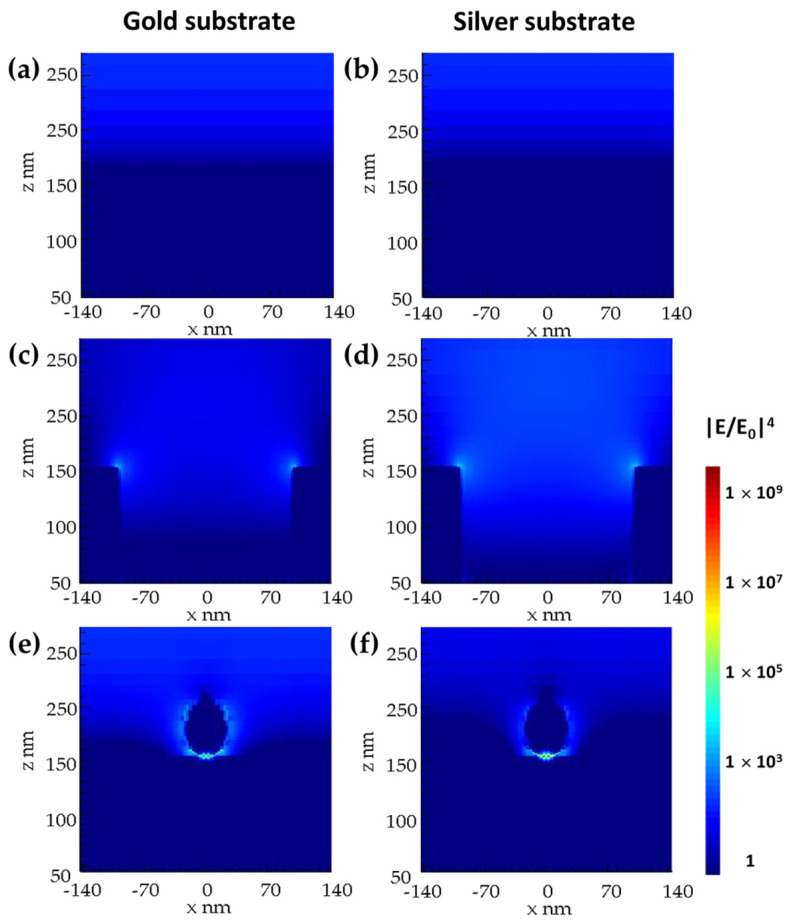
Electric field distribution (|**E**/**E_0_**|^4^) of the electromagnetic Raman enhancement factor for a (**a**) gold and (**b**) silver flat slabs, (**c**) gold and (**d**) silver nanohole arrays, and Ag-NP on (**e**) gold and (**f**) silver slabs.

Figure 4a,b show the simulation result for the electromagnetic enhancement distribution of gold and silver flat slabs with a thickness of 100 nm. For these structures, an electromagnetic enhancement factor (G_SERS_) of 9.3 and 9.0 for both gold and silver, respectively, was obtained. Since there was no generation of hotspots on the surface, the electrons resonating at the same wavelength as the excitation source were the only contributors to Raman enhancement.

Since NHAs and NPs have been demonstrated to have the ability to promote SERS [10,27,41], we investigated the enhancement factor, using the same excitation source, for each of these nanostructures, as they are critical components of the proposed hybrid SERS-active substrate. The e-field distribution and the enhancement factor were calculated for the nanoparticles and the nanohole arrays individually. Figure 4c,d show, respectively, the local e-field distribution for both Au-NHA and Ag-NHA. The region with the highest e-field enhancement is located at the rim of the nanoholes, with a maximum calculated G_SERS_ of 2087 for Au-NHAs and 721 for Ag-NHAs. As the highest e-field enhancement occurs at the EOT maxima [42,43], the highest value for G_SERS_ is found when the excitation source matches the plasmonic resonance in NHAs. Additional FDTD simulations confirmed the highest e-field enhancement of the NHAs plasmonic response in transmission mode (see Supplementary Materials). Au-NHAs presented a higher enhancement factor, since their physical characteristics, such as periodicity and hole size, are optimized for an SPR response at 670 nm, which is closer to a Raman excitation wavelength of 785 nm, compared to the resonance of Ag-NHAs that occur at 660 nm.

Figure 4e,f show, respectively, the e-field distribution of an Ag-NP in contact with flat slabs of gold and silver. The Ag-NP-on-Ag-slab showed an enhancement factor of 1.1 × 10^9^, which is almost two orders of magnitude higher compared to the value of 7.5 × 10^7^ achieved by the Ag-NP-on-Au-slab. In both cases, notably, the maximal e-field confinement is located in the sub-nanometric space between the NP and the slab, adjacent to the contact point, as the plasmonic excitation arises only from the LPSR in the NP. It is hypothesized that the higher response of the Ag-NPS on Ag can be attributed to the fact that the plasmonic response of silver has a higher penetration depth into the dielectric, and higher resonances could be achieved since the optical properties and interaction are the same for the flat and the NP.

### 3.2. Au NHAs: Ag NPs

**Figure 5:**
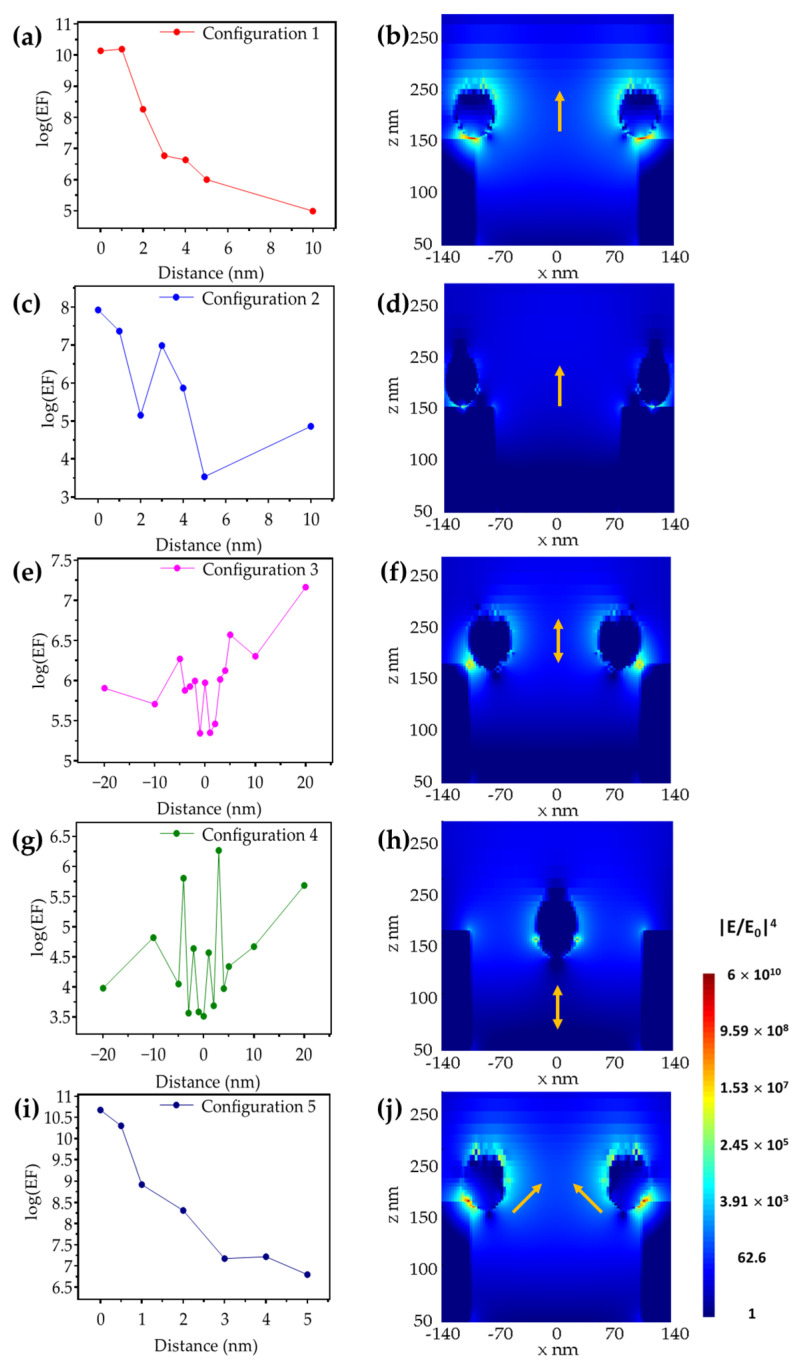
Ag-NP-on-Au-NHA G_SERS_ values for different distances (left column) and e-field distributions (|**E**/**E_0_**|^4^) of the electromagnetic Raman enhancement factor for the position with the highest G_SERS_ (right column) for (**a**,**b**) Configuration 1, (**c**,**d**) Configuration 2, (**e**,**f**) Configuration 3, (**g**,**h**) Configuration 4, and (**i**,**j**) Configuration 5. Yellow arrows indicate the direction in which the particles are displaced.

Due to the enhanced plasmonic resonance response of NHA at 785 nm, we investigated the e-field enhancement of a hybrid structure with Ag-NPs and Au-NHA, considering their potential as SERS-based substrates. The left column in Figure 5 shows the results obtianed from the FDTD simulations for different NP positions relative to the nanoholes. The right column in Figure 5 shows an image of the e-field distribution for the position that resulted in the highest enhancement factor for each configuration. Figure 5a shows the dependence of the magnitude of G_SERS_ on the relative position of the NPs for Configuration 1, while Figure 5b shows the e-field distribution for the position with the highest G_SERS_ with a value of 1.5 × 10^10^, which occurs when the Ag-NP is separated by 1 nm from the rim of the nanoholes along the *z*-axis. The direct contact of the NP with the nanohole rim (i.e., 0 nm in separation) resulted in a G_SERS_ value of 1.3 × 10^10^, which is comparable to the highest value obtained at 1 nm. However, the 1 nm-gap case provides enhancement over a larger volume in the dielectric, which can be equivalent to the spacing provided by capture molecules on a functionalized substrate in a biosensing context. The intensity of the e-field decays with distance, as hypothesized, but it is worth noting that the enhancement factors up to a separation of 5 nm (corresponding G_SERS_ of 1 × 10^6^) are still compatible with SERS applications [32,41]. Figure 5c,d show, respectively, the dependence of the magnitude of G_SERS_ on the relative position of the NPs, as well as the e-field distribution for Configuration 2. In this configuration, the enhancement factor is 8.3 × 10^7^ when the NP is in contact with the surface of the NHA, a similar value compared to the Ag-NP-on-Au-slab case. The NP-NHA configuration shows a skewed e-field distribution due to the proximity of the plasmonic resonance in the NHA, compared to the Ag-NP-on-Au-slab case, where the field distribution is symmetric. 

In Figure 5e, Configuration 3, the perimeter of the nanoparticle is in contact with the inner wall of the nanohole and moves along the vertical axis. In this configuration, the centroid of the NP is displaced 20 nm above and below the nanohole rim. Figure 5f presents the e-field distribution for the case of maximum Raman enhancement, which occurred when the particle was positioned 20 nm above the rim with a corresponding G_SERS_ of 1.4 × 10^7^. The highest intensity of the e-field was confined within a toroidal volume at a distance ~20 nm above the rim, decreasing in magnitude along the inner surface of the nanoholes. Figure 5g shows the enhancement factor variation for Configuration 4, where the particle is located at the center of the nanoholes, and, similar to configuration 3, displaced 20 nm above and below the nanohole rim along the *z*-axis. The separation between the particle and the rim of the nanoholes must be considerable in order to exhibit a strong coupling between the plasmonic resonances of both elements. Nevertheless, when the center of the NP is positioned 3 nm above the edge of the nanoholes, as in Figure 5h, a coupling yields a G_SERS_ value of 1.8 × 10^6^, which is mainly confined to the NP. Finally, Figure 5i shows the G_SERS_ values for different distances corresponding to Configuration 5, when the location of the NPs is 45 degrees from the rim of the nanoholes. Similar to Configuration 1, Configuration 5 exhibited the highest enhancement factor when the NPs were in contact with the NHA surface with a value of 4.7 × 10^10^. Figure 5j shows the e-field distribution for the position with the highest G_SERS._ The high e-field enhancement of this configuration can be attributed to the collective contribution of the plasmonic resonance from the top and the inner wall of the nanohole, which in turn induces a strong LSPR at the top of the particle. 

**Figure 6:**
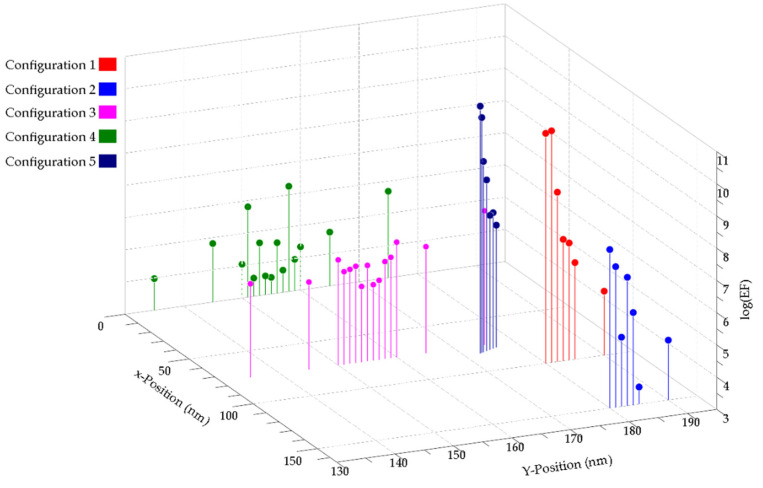
Summary of the Ag-NP-on-Au-NHA G_SERS_ value variation for different positions along the *z*-axis for Configurations 1–5.

Figure 6 presents a summary of the enhancement factor values of all the positions for the five configurations. The highest G_SERS_ values were obtained with Configurations 1 and 5. In particular, Configuration 5 not only yielded the highest enhancement factor value but also showed a large and more homogeneous e-field distribution between the NPs and nanoholes, with the smallest G_SERS_ variation for the distance. Most of the fabrication approaches used to produce SERS-active substrates based on NP assembly have focused on the confinement of e-fields in nanometric and sub-nanometric separation between metallic elements. A spacing of 1 nm or less may relate, in praxis, to the use of small biomolecules such as cysteamine [29] and DNA-assisted assemblies [30] to generate sub-nanometer gaps between metallic structures. Other studies have demonstrated the deposition of monolayers such as graphene [44,45] and MoS_2_ [46] as sub-nanometer spacers between NP-NP structures. In real-world applications, such as the sensing of drugs [47], pollutants [48], and cancer diagnosis [49], having an e-field enhancement over large matrix volumes is highly desirable as it increases the probability of detection [6]. However, having high enhancement values for cases where the particle is touching the surface of the nanohole arrays is not surprising, since it has been demonstrated that a particle touching another metallic surface has the ability to collect and concentrate broadband radiation in the vicinity of the contact point [50,51].

### 3.3. Ag-NHAs: Ag-NPs

**Figure 7:**
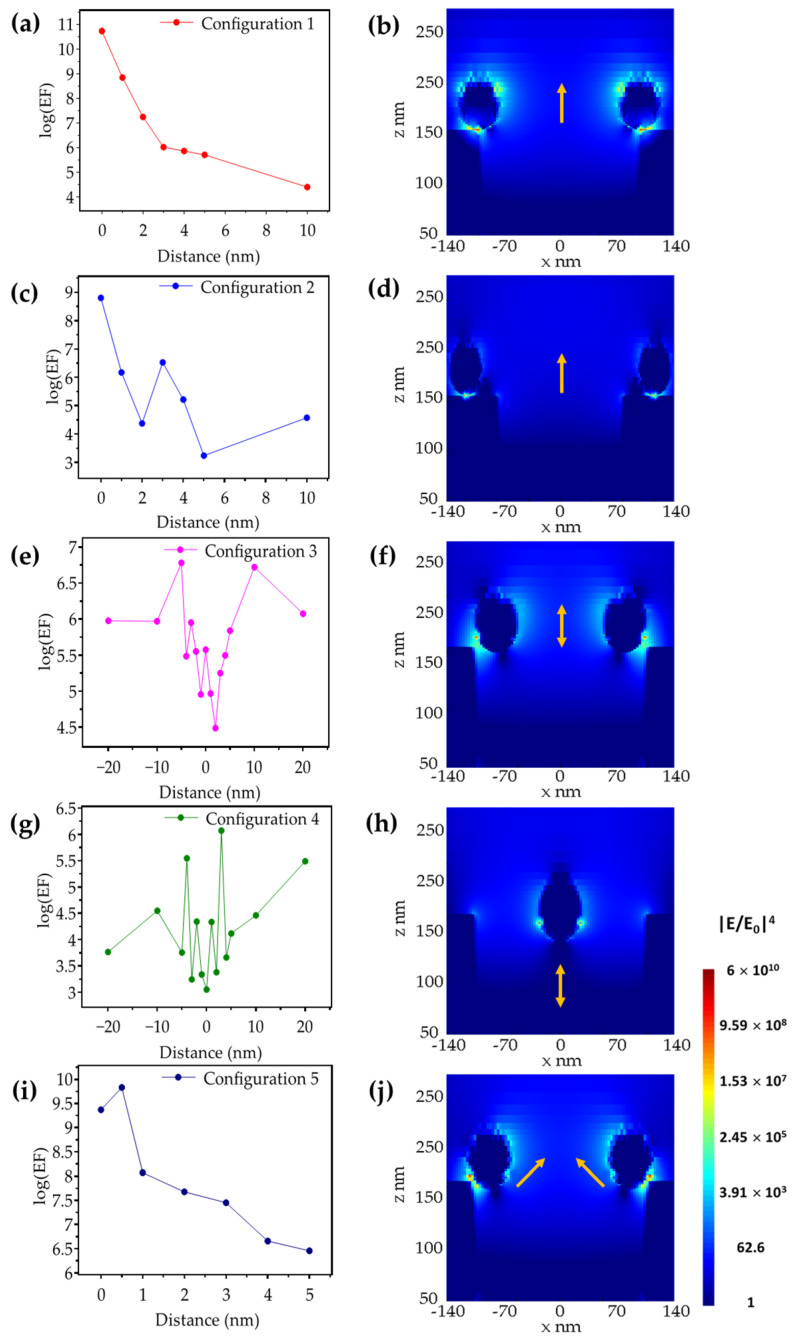
Ag-NP-on-Ag-NHA G_SERS_ values for different distances (left column) and e-field distributions (|**E**/**E_0_**|^4^) of the electromagnetic Raman enhancement factor for the position with the highest G_SERS_ (right column) for (**a**,**b**) Configuration 1, (**c**,**d**) Configuration 2, (**e**,**f**) Configuration 3, (**g**,**h**) Configuration 4, and (**i**,**j**) Configuration 5. Yellow arrows indicate the direction in which the particles are displaced.

Motivated by the high e-field enhancement obtained in the simulations for a Ag-NP-on-Ag-slab when exited by a 785 nm light source, we investigated a hybrid nanostructure formed by Ag-NPs and Au-NHA as a potential SERS-active substrate. Figure 7 presents the results of the simulations carried out for the different configurations of the hybrid Ag-NPs-on-Au-NHA structures. The left column in Figure 7 shows the results obtained from the FDTD simulations for different NP positions relative to the nanoholes, as described in Section 2.2. The right column in Figure 7 shows the image of the e-field distribution for the position that resulted in the highest enhancement factor for each configuration. Specifically, Figure 7a,b show the simulation results obtained for the dependence of the magnitude of G_SERS_ on the relative position of the NPs and the electromagnetic enhancement distribution, respectively, for Configuration 1. The highest G_SERS_ value occurred when the particle was in contact with the rim of the nanoholes, with a value of 5.3 × 10^10^. This enhancement factor is superior to the highest G_SERS_ value obtained from the Ag-NP-on-Au-NHAs analogous nanostructure. However, the intensity of the e-field in configuration 1 for the Ag-NP-on-Ag-NHAs structure decays faster with distance. Configuration 2, presented in Figure 7c,d, shows an enhancement value of 6.3 × 10^8^, which is comparable to the value obtained for the Ag-NP-on-Ag-slab case. A slight but notable skewness of the e-field distribution is observed in this configuration due to the proximity of the plasmonic resonance in the NHA. 

Figure 7e,f show, respectively, the dependence of the magnitude of G_SERS_ on the relative position of the NPs and the e-field distribution for Configuration 3. In this configuration, the perimeter of the nanoparticle is in contact with the inner wall of the nanohole and moves along the vertical axis. Interestingly, the maximum enhancement values occurred when the centroid of the NP was 10 nm above and below the rim, with G_SERS_ values of 5.2 × 10^6^ and 6 × 10^6^, respectively. When the particles were 10 nm above the rim, the resonance was confined within the nanoholes e-field toroidal volume. Contrarily, when the NPs were 10 nm below, the enhancement occurred due to a coupling effect between the particles. In Figure 7g, Configuration 4, it can be seen that the particle is located at the center of the nanoholes in the *x*- and *y*-directions, and it is displaced 20 nm above and below the nanohole rim along the *z*-axis. Similar to Configuration 4 for the Ag-NP-on-Au-NHAs case, the separation between the particle and the rim of the nanoholes must be considerable in order to produce a strong interaction between the plasmonic resonances of both elements. However, there is a weak interaction when the particle, with respect to its center, is positioned 3 nm above the edge of the nanoholes, producing an enhancement factor value of 1.1 × 10^6^ and an e-field distribution as shown in Figure 7h. The lack of axisymmetry between the inner curved region of the nanohole and the upper flat surface leads to an asymmetric enhancement of the electric field that resembles the Fano-like resonances observed in previous studies [52,53]. In Figure 7i, the variation in the G_SERS_ value is shown for different distances for Configuration 5. This configuration exhibited the highest enhancement factor of 6.8 × 10^9^ when the NPs were separated from the edge of the NHAs 0.5 nm at 45 degrees, as shown in Figure 7j. This high e-field enhancement can be attributed to the coupling of the plasmonic resonances between the NPs and the top and inner walls of the nanohole. A quasi-linear trend of the EF is observed in the configurations corresponding to Figure 5a,i and Figure 7a,i, as the gap between the NP and the nanohole is proportionally increased. However, for the rest of the configurations, the gap between the NP and the surface of the nanohole, as well as the position of the surface of the nanoparticle relative to the nanohole rim, varies. The proximity of the NP to the nanohole rim is important due to the high plasmonic activity at the edge of the nanohole and the collective resonance contribution from the top and the inner surfaces. 

**Figure 8:**
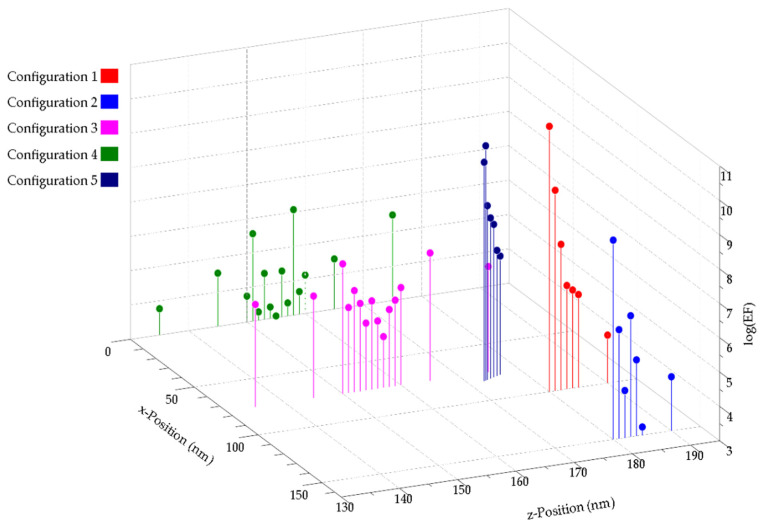
Summary of the Ag-NP-on-Ag-NHA G_SERS_ value variation for different positions in the *z*-axis for Configurations 1–5.

Figure 8 shows a summary of the enhancement factor values of all the positions for the five configurations. In general, the behavior of the electric field distribution between both structures, Ag-NPs-on-Ag-NHA and Ag-NPs-on-Au-NHA, is very similar. For most cases, the enhancement factors obtained for the Ag-NPs-on-Ag-NHA structure are higher when the NP is in contact with the NHA surface, but the G_SERS_ value decreases faster in relation to the distance. In Configuration 5, all the enhancement factor values were higher for the Ag-NPs-on-Au-NHA, and this can be attributed to the higher SPR contribution from the Au-NHAs.

## 4. Conclusions

This work analytically demonstrated the Raman enhancement potential of using Ag-NPs in Ag and Au flow-through NHAs as hybrid SERS-active structures. The FDTD simulations revealed the strong dependency of the electromagnetic enhancement factor and the e-field distribution on the relative position of the nanoparticle with respect to the edge of the rim on the metallic portion of the NHAs. For both structures (i.e., all-silver and gold-silver), the highest enhancement factors were achieved when the NPs were located at 45 degrees from the rim of the nanoholes. For the Ag-NPs-on-Ag-NHA hybrid structure, the maximum G_SERS_ value was 6.8 × 10^9^ and this occurred when the NP was placed 0.5 nm away from the rim of the NHA. For the Ag-NPs-on-Au-NHA hybrid structure, a maximum G_SERS_ value was obtained upon the contact of the NP with the NHA at 4.7 × 10^10^.

Nevertheless, our study indicates that the e-field enhancement achieved for the Ag-NPs-on-Au-NHA hybrid structure reached about 1.7-times larger distances, extending away from the rim of the nanoapertures, compared to that obtained for the Ag-NPs-on-Ag-NHA structure. It is noticeable that the e-field distribution in the former is more symmetric with respect to the rim compared to the latter. Most of the plasmonic activity (i.e., plasmonic enhancement) in the all-silver hybrid structure was found at the upper surface of the NHAs. The intensity, in such a case, also decays more quickly compared to that of the gold–silver structure. Both the symmetry and rapid decay of the field enhancement can be attributed to the optimized SPR response of the Au-NHAs for a 785 nm light source. 

This work provides key information on the impact of the relative spacing between the nanoparticles and the nanoapertures, and the creation of SERS-enabling hotspots between both, when assembled into a single hybrid nanostructure. Future work should focus on harnessing the electromagnetic enhancements of a hybrid nanostructure, which can already benefit from the current reproducible fabrication of large-area plasmonic nanoapertures [54,55], and selective nanoparticle-positioning methods, such as electrokinetics [31].

## Supplementary Materials

The following are available online at https://www.mdpi.com/article/10.3390/bios12020128/s1. Figure S1: Silver NHA FDTD simulations show the (a) transmission spectrum and the (b) Electric field distribution (|**E**/**E**_0_|^4^) of the electromagnetic Raman enhancement factor. Figure S2. Gold NHA FDTD simulations show the (a) transmission spectrum and the (b) Electric field distribution (|**E**/**E**_0_|^4^) of the electromagnetic Raman enhancement factor.

## References

[1] Li X., Soler M., Özdemir C.I., Belushkin A., Yesilköy F., Altug H. (2017). Plasmonic nanohole array biosensor for label-free and real-time analysis of live cell secretion. Lab Chip.

[2] Yanik A.A., Huang M., Kamohara O., Artar A., Geisbert T.W., Connor J.H., Altug H. (2010). An Optofluidic Nanoplasmonic Biosensor for Direct Detection of Live Viruses from Biological Media. Nano Lett..

[3] Gomez-Cruz J., Nair S., Manjarrez-Hernandez A., Gavilanes-Parra S., Ascanio G., Escobedo C. (2018). Cost-effective flow-through nanohole array-based biosensing platform for the label-free detection of uropathogenic *E. coli* in real time. Biosens. Bioelectron..

[4] Nair S., Gomez-Cruz J., Manjarrez-Hernandez A., Ascanio G., Sabat R.G., Escobedo C. (2020). Rapid label-free detection of intact pathogenic bacteria: In situ via surface plasmon resonance imaging enabled by crossed surface relief gratings. Analyst.

[5] Escobedo C., Chou Y.-W., Rahman M., Duan X., Gordon R., Sinton D., Brolo A.G., Ferreira J. (2013). Quantification of ovarian cancer markers with integrated microfluidic concentration gradient and imaging nanohole surface plasmon resonance. Analyst.

[6] Zhao Y., Chu B., Zhang L., Zhao F., Yan J., Li X., Liu Q., Lu Y. (2018). Constructing sensitive SERS substrate with a sandwich structure separated by single layer graphene. Sens. Actuators B Chem..

[7] Nair S., Escobedo C., Sabat R.G. (2017). Crossed Surface Relief Gratings as Nanoplasmonic Biosensors. ACS Sens..

[8] Nair S., Gomez-Cruz J., Manjarrez-Hernandez Á., Ascanio G., Sabat R., Escobedo C. (2018). Selective Uropathogenic E. coli Detection Using Crossed Surface-Relief Gratings. Sensors.

[9] Cetin A.E., Coskun A.F., Galarreta B.C., Huang M., Herman D., Ozcan A., Altug H. (2014). Handheld high-throughput plasmonic biosensor using computational on-chip imaging. Light Sci. Appl..

[10] Dies H., Raveendran J., Escobedo C., Docoslis A. (2017). In situ assembly of active surface-enhanced Raman scattering substrates via electric field-guided growth of dendritic nanoparticle structures. Nanoscale.

[11] Couture M., Brulé T., Laing S., Cui W., Sarkar M., Charron B., Faulds K., Peng W., Canva M., Masson J.-F. (2017). High Figure of Merit (FOM) of Bragg Modes in Au-Coated Nanodisk Arrays for Plasmonic Sensing. Small.

[12] Abdulhalim I. (2014). Plasmonic Sensing Using Metallic Nano-Sculptured Thin Films. Small.

[13] Schlücker S. (2014). Surface-Enhanced Raman Spectroscopy: Concepts and Chemical Applications. Angew. Chem. Int. Ed..

[14] Craig A.P., Franca A.S., Irudayaraj J. (2013). Surface-Enhanced Raman Spectroscopy Applied to Food Safety. Annu. Rev. Food Sci. Technol..

[15] Li Z., Deen M.J., Kumar S., Selvaganapathy P.R. (2014). Raman Spectroscopy for In-Line Water Quality Monitoring—Instrumentation and Potential. Sensors.

[16] Esteso M.A., Ribeiro A.C.F., George S.C., Abraham A.R., Haghi A.K. (2021). Optical and Molecular Physics: Theoretical Principles and Experimental Methods.

[17] Zhang C., Chen S., Jiang Z., Shi Z., Wang J., Du L. (2021). Highly Sensitive and Reproducible SERS Substrates Based on Ordered Micropyramid Array and Silver Nanoparticles. ACS Appl. Mater. Interfaces.

[18] Yue W., Gong T., Long X., Kravets V., Gao P., Pu M., Wang C. (2020). Sensitive and reproducible surface-enhanced raman spectroscopy (SERS) with arrays of dimer-nanopillars. Sens. Actuators B Chem..

[19] Hajshahvaladi L., Kaatuzian H., Danaie M. (2022). A high-sensitivity refractive index biosensor based on Si nanorings coupled to plasmonic nanohole arrays for glucose detection in water solution. Opt. Commun..

[20] Ekşioğlu Y., Cetin A.E., Petráček J. (2016). Optical Response of Plasmonic Nanohole Arrays: Comparison of Square and Hexagonal Lattices. Plasmonics.

[21] Lesuffleur A., Im H., Lindquist N.C., Lim K.S., Oh S.-H. (2008). Laser-illuminated nanohole arrays for multiplex plasmonic microarray sensing. Opt. Express.

[22] De Giacomo A., Lospinoso D., Colombelli A., Lomascolo M., Rella R., Manera M.G. (2022). Self-Assembled Metal Nanohole Arrays with Tunable Plasmonic Properties for SERS Single-Molecule Detection. Nanomaterials.

[23] Nair S., Gomez-Cruz J., Ascanio G., Docoslis A., Sabat R.G., Escobedo C. (2021). Cicada Wing Inspired Template-Stripped SERS Active 3D Metallic Nanostructures for the Detection of Toxic Substances. Sensors.

[24] Yi Z., Liu M., Luo J., Xu X., Zhang W., Yi Y., Duan T., Wang C., Tang Y. (2016). Optical Properties and Local Electromagnetic Field Enhancement of Periodic Rectangular Nanohole Arrays in Au-Interlayer-Au Multilayer Films. Plasmon..

[25] Skehan C., Ai B., Larson S.R., Stone K.M., Dennis W.M., Zhao Y. (2018). Plasmonic and SERS performances of compound nanohole arrays fabricated by shadow sphere lithography. Nanotechnology.

[26] Candeloro P., Iuele E., Perozziello G., Coluccio M.L., Gentile F., Malara N., Mollace V., Di Fabrizio E. (2017). Plasmonic nanoholes as SERS devices for biosensing applications: An easy route for nanostructures fabrication on glass substrates. Microelectron. Eng..

[27] Armas L.E.G., Menezes J.W., Huila M.G., Araki K., Toma H.E. (2016). Gold Nanohole Arrays Fabricated by Interference Lithography Technique as SERS Probes for Chemical Species Such As Rhodamine 6G and 4,4′-Bipyridine. Plasmonics.

[28] Kim J.-Y., Han D., Crouch G.M., Kwon S.-R., Bohn P.W. (2019). Capture of Single Silver Nanoparticles in Nanopore Arrays Detected by Simultaneous Amperometry and Surface-Enhanced Raman Scattering. Anal. Chem..

[29] Seo S., Chang T.-W., Liu G.L. (2018). 3D Plasmon Coupling Assisted Sers on Nanoparticle-Nanocup Array Hybrids. Sci. Rep..

[30] Li L., Wang Z., Lu Y., Zhu K., Zong S., Cui Y. (2021). DNA-assisted synthesis of Ortho-NanoDimer with sub-nanoscale controllable gap for SERS application. Biosens. Bioelectron..

[31] Dies H., Bottomley A., Nicholls D.L., Stamplecoskie K., Escobedo C., Docoslis A. (2020). Electrokinetically-Driven Assembly of Gold Colloids into Nanostructures for Surface-Enhanced Raman Scattering. Nanomaterials.

[32] Le Ru E.C., Blackie E., Meyer M., Etchegoin P.G. (2007). Surface Enhanced Raman Scattering Enhancement Factors:  A Comprehensive Study. J. Phys. Chem. C.

[33] Inan U.S., Marshall R.A. (2011). Numerical Electromagnetics: The FDTD Method.

[34] Kimura W.D. (2017). Electromagnetic Waves and Lasers.

[35] Johnson P.B., Christy R.W. (1972). Optical Constants of the Noble Metals. Phys. Rev. B.

[36] Palik E.D. (1991). Handbook of Optical Constants of Solids.

[37] Philipp H.R. (1973). Optical Properties of Silicon Nitride. J. Electrochem. Soc..

[38] Félidj N., Aubard J., Lévi G., Krenn J.R., Hohenau A., Schider G., Leitner A., Aussenegg F.R. (2003). Optimized surface-enhanced Raman scattering on gold nanoparticle arrays. Appl. Phys. Lett..

[39] Félidj N., Aubard J., Lévi G., Krenn J.R., Salerno M., Schider G., Lamprecht B., Leitner A., Aussenegg F.R. (2002). Controlling the optical response of regular arrays of gold particles for surface-enhanced Raman scattering. Phys. Rev. B.

[40] Kneipp K., Moskovits M., Kneipp K., Moskovits M., Kneipp H. (2006). Surface-Enhanced Raman Scattering.

[41] Langer J., de Aberasturi D.J., Aizpurua J., Alvarez-Puebla R.A., Auguié B., Baumberg J.J., Bazan G.C., Bell S.E.J., Boisen A., Brolo A.G. (2019). Present and Future of Surface-Enhanced Raman Scattering. ACS Nano.

[42] Li Q., Yang Z., Ren B., Xu H., Tian Z. (2010). The relationship between extraordinary optical transmission and surface-enhanced raman scattering in subwavelength metallic nanohole arrays. J. Nanosci. Nanotechnol..

[43] Brolo A.G., Arctander E., Gordon R., Leathem B., Kavanagh K.L. (2004). Nanohole-enhanced raman scattering. Nano Lett..

[44] Zhang C., Li C., Yu J., Jiang S., Xu S., Yang C., Liu Y.J., Gao X., Liu A., Man B. (2018). SERS activated platform with three-dimensional hot spots and tunable nanometer gap. Sens. Actuators B Chem..

[45] Quan J., Zhang J., Li J., Zhang X., Wang M., Wang N., Zhu Y. (2019). Three-dimensional AgNPs-graphene-AgNPs sandwiched hybrid nanostructures with sub-nanometer gaps for ultrasensitive surface-enhanced Raman spectroscopy. Carbon N. Y..

[46] Li Z., Jiang S., Huo Y., Liu A., Zhang C., Yu J., Wang M., Li C., Lu Z., Man B. (2018). 3D Hybrid Plasmonic Nanostructures with Dense Hot Spots Using Monolayer MoS2 as Sub-Nanometer Spacer. Adv. Mater. Interfaces.

[47] Dies H., Raveendran J., Escobedo C., Docoslis A. (2018). Rapid identification and quantification of illicit drugs on nanodendritic surface-enhanced Raman scattering substrates. Sensors Actuators B Chem..

[48] Wang J., Qiu C., Mu X., Pang H., Chen X., Liu D. (2020). Ultrasensitive SERS detection of rhodamine 6G and p-nitrophenol based on electrochemically roughened nano-Au film. Talanta.

[49] Eom G., Kim H., Hwang A., Son H.Y., Choi Y., Moon J., Kim D., Lee M., Lim E.K., Jeong J. (2017). Nanogap-Rich Au Nanowire SERS Sensor for Ultrasensitive Telomerase Activity Detection: Application to Gastric and Breast Cancer Tissues Diagnosis. Adv. Funct. Mater..

[50] Fernández-Domínguez A.I., Maier S.A., Pendry J.B. (2010). Collection and concentration of light by touching spheres: A transformation optics approach. Phys. Rev. Lett..

[51] Noguez C. (2007). Surface Plasmons on Metal Nanoparticles:  The Influence of Shape and Physical Environment. J. Phys. Chem. C.

[52] Cetin A.E., Altug H. (2012). Fano resonant ring/disk plasmonic nanocavities on conducting substrates for advanced biosensing. ACS Nano.

[53] Wu C., Khanikaev A.B., Adato R., Arju N., Yanik A.A., Altug H., Shvets G. (2011). Fano-resonant asymmetric metamaterials for ultrasensitive spectroscopy and identification of molecular monolayers. Nat. Mater..

[54] Kumar S., Cherukulappurath S., Johnson T.W., Oh S.-H. (2014). Millimeter-Sized Suspended Plasmonic Nanohole Arrays for Surface-Tension-Driven Flow-Through SERS. Chem. Mater..

[55] Gong T., Luo Y., Zhao C., Yue W., Zhang J., Zhu Y., Pu M., Zhang Z., Wang C., Luo A.X. (2019). Highly reproducible and stable surface-enhanced Raman scattering substrates of graphene-Ag nanohole arrays fabricated by sub-diffraction plasmonic lithography. OSA Contin..

