# Supplementary material for: FDTD Analysis of Hotspot-Enabling Hybrid Nanohole-Nanoparticle Structures for SERS Detection
Source: Biosensors (Basel). 2022 Feb 17;12(2):128. doi: 10.3390/bios12020128 (PMC8870321; doi:10.3390/bios12020128)
Supplement: Supplementary file 1 [file biosensors-12-00128-s001.zip › biosensors-1583797-supplementary.pdf]

biosensors

MDPI

Supporting information

FDTD Analysis of Hotspot-Enabling Hybrid Nanohole-Nanoparticle Structures for SERS Detection

Juan Gomez-Cruz [1], Yazan Bdour [1], Kevin Stamplecoskie [2] and Carlos Escobedo [1,*]

1. Department of Chemical Engineering, Queen's University, 19 Division St, Kingston, ON K7L 3N6, Canada; j.gomezcruz@queensu.ca (J.G.C.); 16yb6@queensu.ca (Y.B.); ce32@queensu.ca (C.E.)
2. Department of Chemistry, Queen's University, 90 Bader Lane, Kingston, Ontario, K7L 3N6, Canada; kevin.stamplecoskie@queensu.ca (K.S.)

* Correspondence: ce32@queensu.ca; Tel.: +1-613-5333095

FDTD simulation to calculate the transmission spectrum and the electric field distribution were performed for the flow-through Ag-NHAs, Figure S1, and Au-NHAs, Figure S2. Both periodic NHA structures have a 200 nm hole diameter and 400 nm periodicity. The NHA structures are comprised of a 200 nm layer of silicon nitride (Si_3N_4), a 5 nm adhesion layer of chrome (Cr), and a 100 nm layer of gold (Au-NHA) or silver (Ag-NHAs), respectively. Figure S1a shows the transmission spectra of the Ag-NHAs with the highest resonance peak situated at 660 nm. Figure S1b shows the e-field distribution of the Ag-NHAs plasmonic response under an excitation wavelength of 785 nm. The region with the highest e-field enhancement is located at the rim of the nanoholes, with a maximum calculated G_{SERS} of 721. Figure S2a shows the transmission spectra of the Au-NHAs with a resonance peak situated at 670 nm. Figure S2b shows the e-field distribution of the Au-NHAs plasmonic response under an excitation wavelength of 785 nm. The region with the highest e-field enhancement is also located at the rim of the nanoholes, with a maximum calculated G_{SERS} of 2087. The higher enhancement factor of the Au-NHAs is attributed to the proximity of the plasmonic resonance peak to the Raman excitation wavelength of 785 nm.

Citation: Gomez-Cruz, J.; Bdour, Y.; Stamplecoskie, K.; Escobedo, C. FDTD analysis of hotspot-enabling hybrid nanohole-nanoparticle structures for SERS detection. *Biosensors* **2022**, *12*, 128. https://doi.org/10.3390/bios12020128

Received: 19 January 2022
Accepted: 14 February 2022
Published: 17 February 2022

Publisher's Note: MDPI stays neutral with regard to jurisdictional claims in published maps and institutional affiliations.

Copyright: © 2022 by the authors. Licensee MDPI, Basel, Switzerland. This article is an open access article distributed under the terms and conditions of the Creative Commons Attribution (CC BY) license (https://creativecommons.org/licenses/by/4.0/).

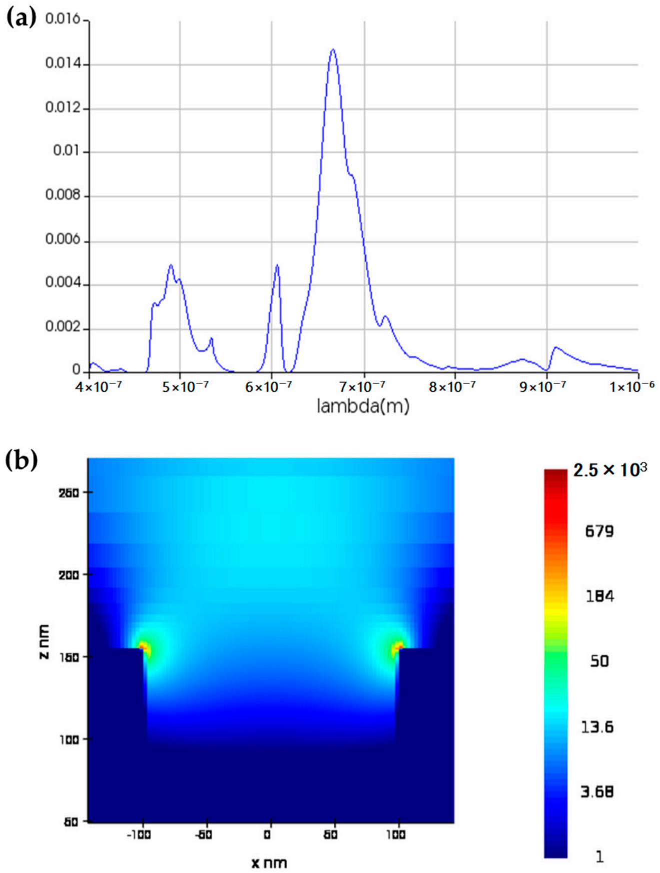

Figure S1. Silver NHA FDTD simulations show the (**a**) transmission spectrum and the (**b**) Electric field distribution ($|\mathbf{E}/\mathbf{E}_0|^4$) of the electromagnetic Raman enhancement factor.$\times 10^{-7}$

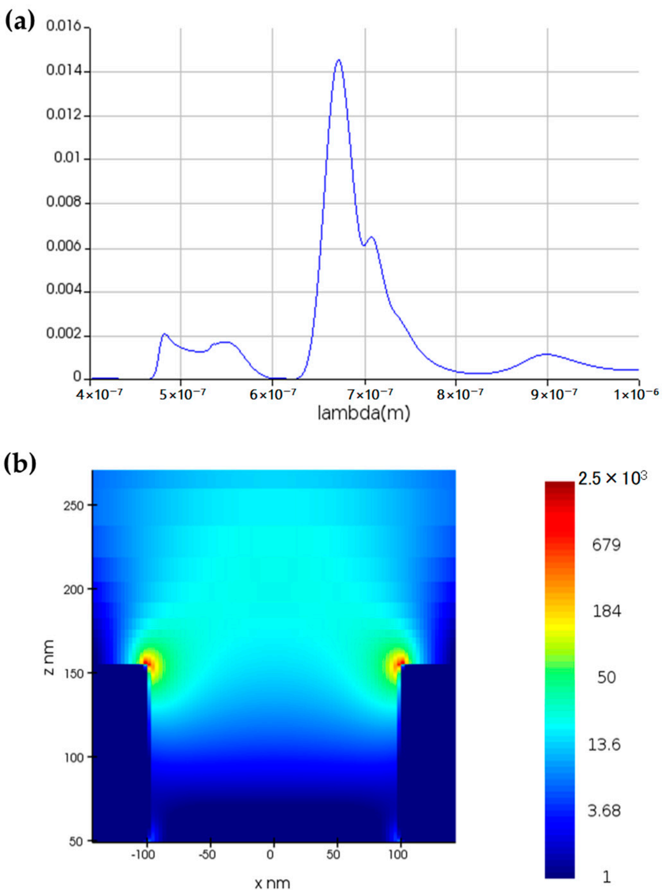

Figure S2. Gold NHA FDTD simulations show the (**a**) transmission spectrum and the (**b**) Electric field distribution ($|\mathbf{E}/\mathbf{E}_0|^4$) of the electromagnetic Raman enhancement factor.
